# The Association between Poor Diet Quality, Physical Fatigability and Physical Function in the Oldest-Old from the Geisinger Rural Aging Study

**DOI:** 10.3390/geriatrics6020041

**Published:** 2021-04-15

**Authors:** Brett Davis, Yi-Hsuan Liu, James Stampley, G. Craig Wood, Diane C. Mitchell, Gordon L. Jensen, Xiang Gao, Nancy W. Glynn, Christopher D. Still, Brian A. Irving

**Affiliations:** 1 Department of Kinesiology, Louisiana State University, Baton Rouge, LA 70803, USA; bdav159@lsu.edu (B.D.); jstamp5@lsu.edu (J.S.); 2 Department of Nutritional Sciences, Pennsylvania State University, University Park, PA 16802, USA; yxl476@psu.edu (Y.-H.L.); dcm1@psu.edu (D.C.M.); xxg14@psu.edu (X.G.); 3 Geisinger Obesity Institute, Geisinger Health System, Danville, PA 17822, USA; cwood@geisinger.edu (G.C.W.); cstill@geisinger.edu (C.D.S.); 4 Larner College of Medicine, University of Vermont, Burlington, VT 05401, USA; gordon.jensen@med.uvm.edu; 5 Graduate School of Public Health, University of Pittsburgh, Pittsburgh, PA 15261, USA; EPIDNWG@pitt.edu; 6 Pennington Biomedical Research Center, Baton Rouge, LA 70808, USA

**Keywords:** nutrition, fatigue, macronutrients, micronutrients, healthy eating index, protein, frailty, physical function, aging, geriatrics

## Abstract

More perceived physical fatigability and poor diet quality are associated with impairments in physical function in older adults. However, the degree to which more perceived fatigability explains the association between poor diet quality and low physical function is unknown. We examined this relationship in 122 (66F, 56M) of the oldest-old participants from the Geisinger Rural Aging Study (GRAS). We used 24-h dietary recalls to assess the Healthy Eating Index (HEI), the Pittsburgh Fatigability Scale (PFS, 0–50) to assess perceived physical fatigability, and the PROMIS Physical Function 20a* to assess physical function. We grouped participants into three age categories: 80–84 (*n* = 51), 85–89 (*n* = 51), and 90+ (*n* = 20) years. Multiple linear regression revealed that a lower HEI was associated with higher PFS Physical score after adjusting for age group, sex, body mass index, and the number of medical conditions (*p* = 0.001). Several macro- and micro-nutrient intakes were also lower in those reporting more (≥15) compared to less (<15) perceived physical fatigability. Mediation analysis revealed that PFS Physical scores explained ~65% (*p* = 0.001) of the association between HEI total score and PROMIS19 Physical Function score. Poor diet quality may contribute to more perceived physical fatigability, which could exacerbate impairments in the oldest-old’s physical function.

## 1. Introduction

Impairments in physical function remain hallmark clinical manifestations of sarcopenia, dynapenia, and frailty in older adults [1,2]. Although impairments in physical function frequently occur with advancing age, the underlying mechanisms contributing to age-related impairments in physical function remain incompletely understood. Moreover, there is considerable inter-individual variability in the degree to which older adults are affected by impairments in physical function, especially among the oldest-old (≥80 years) who now account for ~12.9 million of the United States’ population as of July 1st, 2019 [3]. Additionally, older adults who have a lower self-reported or objectively measured physical function are at increased risk of hospitalization and all-cause mortality [4,5,6]; thus, understanding the mechanisms contributing to impairments in physical function remains clinically relevant. Higher levels of perceived physical fatigue, also known as physical fatigability, was recently shown to be associated with lower levels of physical function, including slower walking speeds during the 400-m walk test [7], and have been shown to predict impending declines in physical function in older adults [8]. Recent results suggest that more perceived physical fatigability is a potential mediator in the disablement pathway [9].

Older adults, particularly the oldest-old, often suffer from poor diet quality and inadequate nutrient intake, leading to subclinical and overt malnutrition [10,11]. Macro- and micronutrient deficiencies are increasingly common in malnourished independent-living older adults (5%–10%), institutionalized older adults (30%–60%), and hospitalized older patients (35%–65%) [12]. Suboptimal micro- and macro-nutrient intake likely contributes to age-related declines in muscle mass, strength, and quality [13,14,15] and may, directly or indirectly, contribute to impairments in physical function, mobility, and the development of sarcopenia, dynapenia, and physical frailty in older adults [2,16,17,18,19]. There is growing evidence that suggests insufficient protein intake could be partially responsible for these age-related deficits [20]. Recent data indicate that the recommended daily allowance (RDA) of protein (0.8 g/kg of body weight/day) may not be sufficient for older adults, and a recent review suggests that those with severe illness or evident malnutrition may need up to 2 g/kg of body weight/day [20]. Although the impact that poor diet quality has on perceived physical fatigability in the oldest-old remains mostly unexplored, recent evidence suggests that unexplained weight loss, a clinical indicator of malnutrition in older adults [21], is associated with moderate and severe fatigue in patients recently discharged from a geriatric hospital [22]. Recent data also suggest that higher diet quality, as reflected by a higher Mediterranean diet score, is associated with a lower risk of frailty in 192 community-dwelling older (>75 years) adults [23]. Poor diet quality can lead to the inadequate intake of macronutrients, micronutrients, or both, which likely contributes to both higher perceived physical fatigability and lower physical function. However, studies are needed that examine whether the association between poor diet quality and lower physical function is explained through higher levels of perceived physical fatigability.

In this paper, we first quantified self-reported diet quality and physical function in a subset of the oldest-old (≥80 years) participants from the Geisinger Rural Aging Study (GRAS) cohort [24] who also reported either high or low levels of physical fatigability. Next, we examined the independent associations between diet quality on physical fatigability and diet quality and physical function. Finally, we used mediation analysis to examine the potential mediating effects of higher physical fatigability on the association between poor diet quality and self-reported physical function. Perceived physical fatigability was assessed using the Pittsburgh Fatigability Scale [25], self-reported physical function was assessed using the PROMIS Physical Function Short-Form 20a, and diet quality was assessed using 24-h diet recalls and the Healthy Eating Index (HEI) [26]. We hypothesized that poor diet quality assessed by the HEI would be associated with more perceived physical fatigability and lower self-reported physical function. We hypothesized that lower protein intake and a lower intake of bioactive micronutrients would also be associated with more perceived physical fatigability as well as lower self-reported physical function. Finally, we hypothesized that more perceived physical fatigability would mediate some of the association between poor diet quality and low self-reported physical function.

## 2. Materials and Methods

### 2.1. Study Population

The Geisinger Rural Aging Study (GRAS) is a longitudinal cohort of predominately white, non-Hispanic older adults residing in rural Pennsylvania [24]. The original cohort of 21,645 was recruited in 1994 when they were ≥65 years of age from the Geisinger Health System and described in detail [24]. All participants in the original cohort provided the Geisinger Institutional Review Board (IRB) informed written consent before their participation, which included longitudinal follow-up [24]. For the present study, we recruited participants from a subset (*n* = 1556) of the original GRAS cohort who were ≥80 years of age between 2015 and 2016 [27]. Potentially eligible participants were pre-screened using a Geisinger IRB-approved electronic medical record (EMR) review [27]. Participants who met the inclusion criteria and did not have an ICD9 code indicating the presence of clinically diagnosed dementia were contacted following this pre-screening (*n* = 1201) [27]. Final screening instruments and questionnaires were administered to participants who provided Geisinger IRB-approved consent by telephone before enrolling in this study (*n* = 174) [27]. Potential participants were excluded if they were identified as having dementia based on the Mini-Mental State Exam (MMSE) [28] or severe depression based on the Geriatric Depression Scale (GDS) [29]. Finally, after excluding participants with incomplete dietary data, 122 participants remained for subsequent analysis, as previously reported [27]. Supplemental Table S1 presents the demographic and health-related characteristics of the GRAS cohort participants who were potentially eligible, eligible but did not participate in the present study, and those who completed the present study.

### 2.2. Demographic and Health-Related Characteristics

A Geisinger IRB-approved EMR review was performed to assess the following demographic and health-related characteristics: height, weight, fasting lipids, and blood glucose [27]. The EMR was also reviewed for the presence of chronic medical conditions, including diabetes, cardiovascular disease, hypertension, obstructive sleep apnea, depression, and osteoarthritis, based on ICD9 codes [27].

### 2.3. Questionnaire Assessments

Participants completed structured phone interviews during which trained research personnel administered the following questionnaires: the Pittsburgh Fatigability Scale (PFS) [25], PROMIS Short Form v1.0—Physical Function 20a* Questionnaire [30]. The PFS Physical score could range from 0 (least severe fatigability) to 50 (most severe fatigability) [25]. As previously established [31,32], we categorized participants by perceived fatigability status (more (PFS Physical scores ≥ 15); less (PFS Physical scores < 15)). The PROMIS Physical Function 20a* raw-score can range from 20–100 (lower scores = worse function). For the present analysis, one question, question pfa11, was excluded from the PROMIS19 Physical Function score due to inadvertent omission of the question during the structured telephone interviews. The present PROMIS19 Physical Function scores could range from 19–95 (low physical function to high physical function).

### 2.4. Twenty-Four-Hour Dietary Recalls

Participants completed a total of three twenty-four-hour dietary recalls to assess their usual dietary intake. Specifically, the twenty-four-hour dietary recalls were collected on three unannounced, randomly selected days, which included two non-consecutive weekdays and one weekend day using the Nutrition Data System for Research (NDSR 2015 and 2016, Nutrition Coordinating Center, University of Minnesota, MN) by trained personnel in the Pennsylvania State University Diet Assessment Center [27]. All twenty-four-hour recalls were conducted by telephone. Nutrient and food group data were generated using NDSR 2016 and were re-expressed as either cup or ounce equivalents per 1000 kcal [27]. Additionally, fatty acid intakes were expressed as ratios [27]. The nutrient and food group data were then used to calculate the Healthy Eating Index 2015 scores [33]. The HEI scores were calculated based upon adequacy and moderation of 13 dietary components and could range from 0 to 100, as previously described [33]. Higher HEI scores represent better adherence to the Dietary Guidelines for Americans [33].

### 2.5. Statistical Analysis

JMP Pro 14 (SAS Institute, Cary, NC, USA) was used to perform statistical analyses. Data are presented either mean ± SD for unadjusted values or as covariate-adjusted means ± SE unless otherwise noted. HEI calculations were conducted in SAS version 9.4 (SAS Institute, Cary, NC, USA) using the methodology and SAS code provided by the Nutrition Coordinating Center (University of Minnesota, Minneapolis, MN, USA) [27].

Student’s T-tests were used to assess mean differences between individuals reporting more compared to less perceived physical fatigability for the unadjusted continuous variables. The log-likelihood ratio tests were used to assess differences between individuals by perceived fatigability status for categorical variables. Multivariable regression analyses were used to test the association between the individual macro- and micronutrient data in participants reporting either more versus less perceived physical fatigability adjusting for the age group (80–84, 85–89, 90+ years), sex (M vs. F), body mass index (BMI), the total number of medical conditions (0–7), and energy intake. Multivariable regression analyses were used to assess the association between HEI and PFS Physical scores after adjusting for the age group (80–84, 85–89, 90 + years), sex (M vs. F), BMI, and the total number of medical conditions (0–7). Leverage plots were used to visually display the effect of the HEI on the PFS Physical and PROMIS19 Physical Function scores after adjusting for the age group (80–84, 85–89, 90+ years), sex (M vs. F), BMI, and the total number of medical conditions (0–7).

We used a mediation analyses approach to test our hypothesis that more perceived physical fatigability explained some of the association between poor diet quality and lower physical function. The mediation analysis was performed using SAS9.4 “proc causalmed”. The dependent (outcome) variable was set as the PROMIS19 Physical Function score, the independent variable was set as the HEI total score, and the mediating variable was set as the PFS Physical scores. The model was also adjusted for the age group, sex, BMI, and the total number of medical conditions. The 95% confidence intervals (95% CI) were based on bootstrapping (1000 samples). A similar mediation analysis was performed using the HEI protein score as the independent variable rather than the HEI total score.

## 3. Results

### 3.1. Demographic and Health-Related Characteristics in Individuals Reporting More vs. Less Perceived Physical Fatigability

The current subset of the oldest-old adults from the GRAS cohort was between 82 and 97 years of age and consisted of 54% women [27]. The unadjusted PFS Physical score was 23 ± 10. Table 1 presents the overall demographic and health-related characteristics stratified by more (≥15) vs. less (<15) perceived physical fatigability. The overall percentage of participants reporting more physical fatigability was ~80%. There was a non-significant trend in those reporting more than less perceived physical fatigability to consist of a higher percentage of 90 + -year-old participants (*p* = 0.097) and a higher percentage of females (*p* = 0.069). We also observed lower PROMIS19 Physical Function score in the participants reporting more compared to less physical fatigability (~12.1% lower, *p* < 0.0001, Table 1). There were no other statistically significant differences in the prevalence (%) of several age-related chronic medical conditions by perceived physical fatigability status (all *p* > 0.05).

### 3.2. Healthy Eating Index Data by Fatigability Status

Table 2 presents the HEI summary data stratified by the presence of more versus less perceived physical fatigability after adjusting for age, sex, BMI, and the total number of medical conditions. The HEI was also lower in those reporting more compared to less physical fatigability (11.7% lower, *p* = 0.028, Table 2). The HEI total protein and seafood and plant protein subscores were significantly lower in the participants reporting more compared to less physical fatigability (all *p* < 0.05, Table 2). Furthermore, the HEI greens and beans subscore was also significantly lower in the participants reporting more compared to less physical fatigability (*p* = 0.019, Table 2).

### 3.3. Macro- and Micronutrient Intake Data by Fatigability Status

There was no significant difference in the energy intake between the participants reporting more compared to less physical fatigability (1485 ± 41 kcals/day vs. 1528 ± 85 kcals/day, *p* = 0.633) adjusting for age, sex, BMI, and the total number of medical conditions. Table 3 presents the macro- and micronutrient intakes stratified by the presence of more versus less perceived physical fatigability after adjusting for age, sex, BMI, the total number of medical conditions, and total energy intake. Lower intake of several macro- and micronutrients was noted in the individuals reporting more perceived physical fatigability. Specifically, total protein, fiber, Vitamin A, Vitamin K, Vitamin B6, Mg^++^, Zn^++^, Mn^++^, and phosphorous intakes were all lower in the individuals reporting more compared to less physical fatigability (all *p* < 0.05, Table 3). Supplemental Table S2 presents the unadjusted macro- and micronutrient intake data stratified by physical fatigability status for additional reference.

### 3.4. PFS Physical Score by HEI Total Score

**Figure 1:**
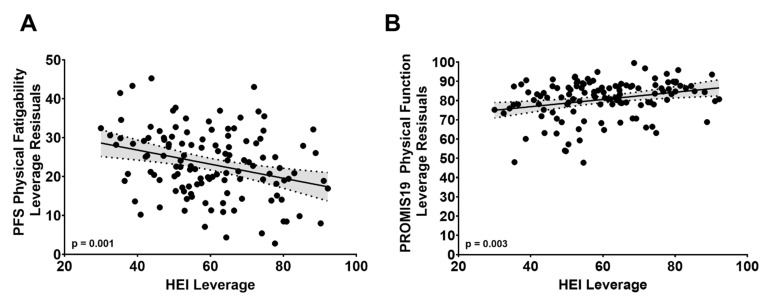
Leverage plots displaying the effect of the Healthy Eating Index (HEI) on Pittsburgh Fatigability Scale (PFS) Physical score (**A**) and PROMIS19 Physical Function (**B**) score after adjusting for age, sex, BMI, and the total number of medical conditions in a subset of 122 oldest-old participants from the Geisinger Rural Aging Study.

Univariate analysis revealed that PFS Physical score was inversely associated with the HEI total score (r = −0.27, *p* = 0.0026). Multiple regression analysis further revealed that a 10-unit lower HEI total score was associated with a 1.8-unit (±0.5 standard error) higher PFS Physical scores (*p* = 0.001) after adjusting for the age group, sex, BMI, and the total number of medical conditions. Figure 1A displays the negative association between the HEI total score and PFS Physical scores after adjusting for age group, sex, BMI, and the total number of medical conditions using a Leverage plot.

### 3.5. PROMIS19 Physical Function Score by HEI Total Score

Univariate analysis revealed that the PROMIS19 Physical Function score was positively associated with the HEI-Total score (r = +0.29, *p* = 0.0012). Multiple regression analysis also revealed that a 10-unit lower HEI score was associated with a 1.9-unit (±0.6 standard error) lower PROMIS19 Physical Function score (*p* = 0.003) after adjusting for the age group, sex, BMI, and the total number of health conditions. Figure 1B is the Leverage plot displaying the positive association between the HEI and PROMIS19 Physical Function score after adjusting for age group, sex, BMI, and the total number of health conditions.

### 3.6. PFS Physical Score by PROMIS19 Physical Function Score

Univariate analysis revealed that a higher PFS Physical score was associated with a lower PROMIS19 Physical Function score (r = −0.65, *p* < 0.0001).

### 3.7. Mediation Analysis

**Figure 2:**
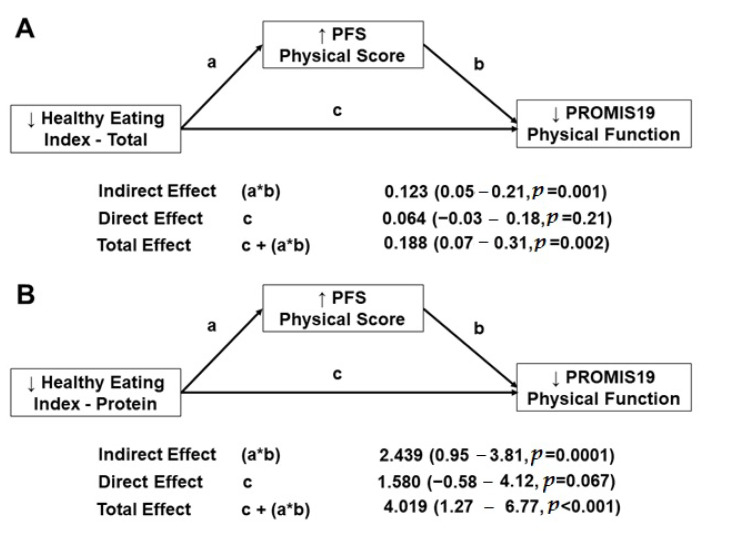
(**A**) shows the statistical mediation analysis for the associations of the lower Healthy Eating Index, more self-reported physical fatigability, on lower self-reported physical function through in a subset of 122 oldest-old participants from the Geisinger Rural Aging Study. (**B**) shows the statistical mediation analysis for the associations of the lower Healthy Eating Index—protein, more self-reported physical fatigability, on lower self-reported physical function through in a subset of 122 oldest-old participants from the Geisinger Rural Aging Study. The dependent (outcome) variable was set as the PROMIS19 Physical Function score, while the independent variable was set as the HEI total (**A**) or HEI protein (**B**), and the mediating variable was set as the Pittsburgh Fatigability Scale (PFS) Physical score. The models were also adjusted for the age group, sex, BMI, and the total number of medical conditions. The 95% confidence intervals (95% CI) were based on bootstrapping (1000 samples).

We found that the HEI total score (independent variable) had a total effect (β = 0.188, 95% CI = 0.07–0.31, *p* = 0.002) on the PROMIS19 Physical Function score (dependent variable) (Figure 2A). We also found that the HEI total score had a natural indirect effect (β = 0.123, 95% CI = 0.05–0.21, *p* = 0.001) on the PROMIS19 Physical Function score, indicating that the PFS Physical score (mediating variable) explained ~65% (*p* = 0.001) of the association between the HEI total score and the PROMIS19 Physical Function score. Finally, after accounting for the PFS Physical score’s mediating effect on this relationship, the HEI total score’s natural direct effect on the PROMIS19 Physical Function score was not significant.

We also found that the HEI protein score (independent variable) had a total effect (β = 4.019, 95% CI = 1.27–6.77, *p* < 0.001) on the PROMIS19 Physical Function score (dependent variable) (Figure 2B). In addition, the HEI protein score had a natural indirect effect (β = 2.439, 95% CI = 0.95–3.81, *p* = 0.0001) on the PROMIS19 Physical Function score, indicating that the PFS Physical score (mediating variable) explained ~61% (*p* < 0.0001) of the association between the HEI protein score and the PROMIS19 Physical Function score. Finally, after accounting for the PFS Physical score’s mediating effect on this relationship, the HEI protein score’s natural direct effect on the PROMIS19 Physical Function score was not significant.

## 4. Discussion

The current study assessed the association between diet quality, self-reported perceived physical fatigability, and physical function in a subset of the oldest-old (≥80 years) participants from the GRAS. Our study’s primary findings are, first, the diet quality, assessed by the HEI, was lower in the participants that reported more compared to less perceived physical fatigability. As expected, lower protein intake levels were also reported in the participants with more compared to less perceived physical fatigability. We also identified several vitamins (e.g., A, B, and K) and metabolically active minerals (Mg^++^, Zn^++^, Cu^++^, Mn^++^, and phosphorous) that were taken in at lower levels in the participants that reported higher compared to lower perceived physical fatigability. Since self-reported physical function, assessed by the PROMIS19 Physical Function score, was lower in the participants who reported more compared to less perceived physical fatigability, we performed a mediation analysis to assess whether higher PFS Physical scores explained the association between poor diet quality and lower self-reported physical function. Our mediation analysis revealed that higher PFS Physical scores significantly explained the association between poor diet quality and lower self-reported physical function.

Although there has been considerable interest in the association between poor diet quality and reduced physical function in older adults [34,35,36,37], the association between poor diet quality and perceived physical fatigability in older adults remains unexplored. Consistent with the studies mentioned above, the HEI total scores were positively associated with the PROMIS19 Physical Function scores in the present study (Figure 1B). Of interest, the HEI total score was lower in participants who reported more compared to less perceived physical fatigability. Although the underlying mechanisms by which lower diet quality contributes to more perceived physical fatigability is unknown, as mentioned above, there is emerging evidence that poor dietary intake is also associated with impairments in physical function in older adults. Thus, one may postulate that perceived physical fatigability is on the pathway between poor dietary intake and impairments in physical function. Therefore, we tested this hypothesis using mediation analysis. Indeed, ~65% of the association between the HEI total score and the PROMIS19 Physical Function score was explained by the PFS Physical score. Future prospective studies are needed to test the potential mediating effect of physical fatigability on the association between poor dietary quality and impaired physical function in older adults.

Subclinical and clinical protein malnutrition is increasingly common in older adults, especially among the oldest-old [38]. Suboptimal protein intake is often associated with reduced physical function in older adults [39,40]. Moreover, the present results revealed that protein intake was lower in the participants who reported more compared to less perceived physical fatigability. Moreover, ~61% of the association between the HEI protein score and the PROMIS19 Physical Function score was explained by the PFS Physical score. Our cohort’s average protein intake was ~0.8 g/kg/d, which included ~56% of the participants having protein intakes below the RDA cut-point (0.8 g/kg/d). The proportion of our participants that reported protein intakes below the RDA cut-point is higher in our cohort than the proportion observed in a slightly younger cohort of community-dwelling older (>65 years) adults (~56% vs. 10%) [41]. Moreover, participants in the Newcastle 85+ study also showed low protein consumption in 28% of their similarly aged cohort of community-dwelling older adults [42]. One potential reason for our higher percentage of participants reporting low protein intake compared to the Newcastle 85+ study could be due to differences in food accessibility in rural environments compared to more urban environments. The low protein intakes are particularly problematic considering that there is increasing consensus that older adults should consume higher protein intakes (e.g., >1.2 g/kg/d) [43] than recommended by the RDA due to the presence of anabolic resistance [44].

The low dietary intake of metabolically active minerals, including Mg^++^, Zn^++^, Cu^++^, Mn^++^, and phosphorous, are frequently observed in age-related skeletal muscle and neurological conditions [45]. In the present study, lower total dietary intake (mg) of Mg^++^, Cu^++^, Mn^++^, and phosphorous were reported in the participants who reported more compared to less perceived physical fatigability. Although there is limited data that directly assess the associations between dietary intake of these metabolically active minerals and perceived physical fatigability in older adults, previous studies have shown links between their lower intakes or deficiencies with impairments in physical function and frailty in older adults [46,47]. Magnesium is well known for its role as a co-factor in many enzymatic reactions, including its essential role in the adenosine triphosphate (ATP) metabolism (mg-ATP). Thus, deficiencies in Mg^++^ will likely result in reductions in skeletal muscle and neurological function due to the high energy demand of these metabolically active tissues, contributing to more perceived fatigability. Along these lines, data from the InCHIANTI study revealed that low circulating concentrations of Mg^++^ were associated with suboptimal skeletal muscle performance in community-dwelling older adults (*n* = 1453, ~67 years of age) [46]. Of note, regardless of perceived physical fatigability status, less than 5% of the current participants met the daily recommended intake of Mg^++^. A recent review highlights the potential detrimental impacts that low circulating Mg^++^ levels have on the elderly and suggests that targeted dietary modifications may be necessary for older adults to achieve optimal Mg^++^ levels in the elderly [48]. Copper deficiencies are often associated with perceptions of fatigue, which may be due to the vital role Cu^++^ plays in heme biosynthesis [49,50]. Although the cause of phosphate deficiency (hypophosphatemia) in the elderly is not well understood, preclinical data suggest that the ability of a muscle to produce ATP may be impaired [51], which could exacerbate perceptions of fatigue in older adults. We should also recognize that lower dietary intakes of these metabolically active minerals may not have manifested in lower circulating concentrations.

Our findings also revealed lower intakes of several essential vitamins, including vitamins A, K, and B_6_, in the participants who reported more compared to less perceived physical fatigability. Lower intake of these essential vitamins could contribute to more perceived physical fatigability and concomitantly lower self-reported physical function. Consistent with the present findings, recent data from the Seniors-ENRICA study, a cohort of ~1630 community-dwelling adults aged ≥60 years, also reported a higher risk of frailty in those reporting low vitamin B_6_ intake [52]. Of interest, the vitamin B_6_ intake in their frail participants, although low (1.9 mg/day), was higher than the vitamin B_6_ intake in our subjects, reporting higher perceived physical fatigability (1.5 mg/day). Recent data from the same cohort also suggest lower odds for mobility impairment in the participants who were in the highest compared to the lowest tertile of Vitamin B_6_ intake (*p* = 0.05) [53]. Seafood is an excellent source of Vitamin B_6_ [54]. Interestingly, the HEI seafood and plant protein subscores were also lower in those reporting more compared to less perceived physical fatigability. A recent report also suggested that higher seafood intake was associated with faster gait speeds in older (65+ year) Norwegian females [55]. Additionally, consistent with the present findings, data from the Health Aging and Body Composition Study suggest that higher Vitamin K intake is associated with better lower extremity physical function in community-dwelling older adults [56]. Considering that green leafy vegetables are excellent sources of Vitamin K and our subjects who reported a lower HEI greens and beans subscore also reported more compared to less perceived physical fatigability is not surprising. To our knowledge, this is the first study to report lower Vitamin A intake in older adults by perceived fatigability status. Future studies are warranted to assess the biological relevance of the association between low Vitamin A intake on physical fatigability.

These oldest-old participants from the GRAS cohort primarily live in rural communities, which makes them potentially vulnerable to poor diet quality. As indicated above, several macro- and micro-nutrient intakes were just at or below RDA recommendations, which could have blunted the association between poor diet quality and perceived physical fatigability. Moreover, ~80% of our participants were identified as having more perceived physical fatigability, which could have blunted some of the association between poor diet quality and perceived physical fatigability. Recent data from the Long Life Family Study also showed a similarly high prevalence (~78%) of oldest-old (80 + -year-olds, *n* = 518) participants who reported more perceived physical fatigability [32]. Moreover, the Long Life Family Study showed that physical function was also lower in the oldest-old participants who reported greater perceived physical fatigability [32]. These two studies show that most of the oldest-old are likely affected by greater perceived physical fatigability, which could mediate some of the association between poor diet quality and reduced physical function in the oldest-old. Future studies are warranted to assess this mediating effect further.

A strength of the present study is, for the first time, we link comprehensive dietary intake data with perceived physical fatigability and self-reported physical function in adults over the age of 80 years. The inclusion of three unannounced randomly selected twenty-four-hour recalls that comprised a total of two non-consecutive weekdays and one weekend day provides a more representative assessment of usual dietary intake and confers more validity to the present findings. The mediation analysis, which identified physical fatigability as a significant mediator of the association between diet quality on self-reported physical function, further strengthens this study. Another strength of the study was that our cohort was evenly distributed between males and females. We also were able to adjust our models based on the number of chronic medical conditions, to account for the potential confounding effect of chronic disease on perceptions of physical fatigability. One limitation of the present study is that the dietary intake data were self-reported, which could be prone to the under-reporting of intake, which could have further exacerbated age-related memory issues. We also recognize that screening out participants who demonstrated dementia or severe depression could also result in weaker associations between dietary intake and the perceptions of fatigability. We would also like to note that the first 14 participants completed their assessments in person, while the remaining 108 participants completed all assessments by telephone. The inadvertent omission of one question from the PROMIS Physical Function 20a questionnaire during the telephone interviews could have potentially affected the PROMIS Physical Function Score’s validity. We also acknowledge that the present study’s results were observed in a predominately non-Hispanic, white population from rural Pennsylvania, who were free of overt dementia and were over 80, which limits the generalizability of our findings. However, as noted above, the participants in the present study, similar to the oldest-old in the Long Life Family Study, showed that physical function was also lower in the oldest-old participants who reported more perceived physical fatigability [32]. Finally, the high rate of exclusion contributed to the small sample size. 

## 5. Conclusions

In summary, overall diet quality, assessed by the HEI, was lower in the oldest-old participants from the GRAS participants who reported more perceived physical fatigability. The intakes of protein, greens and beans, and several macro- and micro-nutrients were also lower in participants who reported more compared to less perceived physical fatigability. As expected, those with higher PFS Physical scores had lower self-reported physical function. Thus, poor diet quality and low intakes of some macro- and micronutrients may contribute to more perceived physical fatigability in the oldest-old, which could further exacerbate impairments in physical function and lead to increased risk of frailty and reduced quality of life. Indeed, our mediation analysis revealed that physical fatigability significantly explained the effect of poor diet quality on self-reported physical function in our cohort. Future work is needed to determine whether targeted improvements in diet quality and intake can improve perceptions of physical fatigability and self-reported physical function.

## Figures and Tables

**Table 1:** Demographic and health-related characteristics data in a subset of 122 oldest-old participants from the Geisinger Rural Aging Study according to perceived physical fatigability status: Pittsburgh Fatigability Scale (PFS).

Characteristics	Physical Fatigability Status		
	More, ≥15	Less, <15	*p*-Value
*n*	98	24	
Age			0.097
80–84 years, %	38.8	54.2	
85–89 years, %	41.8	41.7	
90+ years, %	19.4	4.2	
Female, %	58.2	37.5	0.069
History of diabetes, %	27.6	16.7	0.256
History of coronary artery disease, %	46.9	33.3	0.224
History of hypertension, %	84.7	83.3	0.870
History of obstructive sleep apnea, %	15.3	12.5	0.724
History of depression, %	3.1	4.2	0.791
History of osteoarthritis, %	56.1	41.7	0.203
History of liver disease, %	1.0	0.0	0.507
Body Mass Index (BMI, kg/m^2^), mean ± SD	28.6 ± 4.9	27.9 ± 4.8	0.490
PROMIS Physical Function, mean ± SD	78.5 ± 11.4	89.3 ± 5.1	<0.0001

*p*-values for unpaired T-Tests assuming unequal variance for continuous variables or for the *X*^2^ using the likelihood ratio test.

**Table 2:** Adjusted Healthy Eating Index (HEI) data in a subset of 122 oldest-old participants from the Geisinger Rural Aging Study according to perceived physical fatigability status: Pittsburgh Fatigability Scale (PFS).

Characteristics	Physical Fatigability Status		
	More, ≥15	Less, <15	*p*-Value
*n*	98	24	
HEI	59.0 ± 1.6	66.8 ± 3.3	0.028
HEI-Total Vegetables	3.5 ± 0.1	4.1 ± 0.3	0.066
HEI-Greens and Beans	1.8 ± 0.2	3.0 ± 0.4	0.019
HEI-Total Fruits	3.1 ± 0.2	3.5 ± 0.4	0.357
HEI-Whole Fruits	3.6 ± 0.2	3.9 ± 0.4	0.459
HEI-Whole Grains	5.6 ± 0.4	6.1 ± 0.7	0.467
HEI-Total Dairy	6.5 ± 0.3	6.6 ± 0.6	0.850
HEI-Total Protein	4.3 ± 0.1	4.8 ± 0.2	0.027
HEI-Seafood and Plant Protein	2.7 ± 0.2	3.7 ± 0.4	0.035
HEI-Fatty Acids	3.5 ± 0.3	4.0 ± 0.7	0.522
HEI-Sodium	5.6 ± 0.3	5.7 ± 0.7	0.927
HEI-Refined Grains	6.4 ± 0.4	7.5 ± 0.8	0.204
HEI-Added Sugars	7.1 ± 0.3	7.9 ± 0.6	0.230
HEI-Saturated Fats	5.2 ± 0.4	6.1 ± 0.7	0.298

Data are covariate-adjusted mean ± SE. Covariates included age group, sex, BMI, and total number of medical conditions. *p*-values for mean differences in the individual HEI scores between fatigability categories after adjusting for covariates.

**Table 3:** Adjusted macro- and micronutrient intake data in a subset of 122 oldest-old participants from the Geisinger Rural Aging Study according to perceived physical fatigability status: Pittsburgh Fatigability Scale (PFS).

Characteristics	Physical Fatigability Status		
	More, ≥15	Less, <15	*p*-Value
*n*	98	24	
Fat, g	55.6 ± 1.1	53.5 ± 2.2	0.376
Carbohydrates, g	193.0 ± 3.1	194.0 ± 6.3	0.885
Protein, g	57.1 ± 1.3	64.7 ± 2.7	0.010
Fiber, g	16.4 ± 0.6	19.9 ± 1.2	0.007
Vit A, μg RAE	677.7 ± 30.3	814.7 ± 62.4	0.044
Vit D, μg	4.8 ± 0.3	5.4 ± 0.7	0.379
Vit E, mg AT	8.2 ± 0.6	8.8 ± 1.2	0.671
Vit K, μg	75.3 ± 11.7	130.0 ± 24.0	0.037
Vit B_6_, mg	1.5 ± 0.1	1.9 ± 0.1	0.001
Vit C, mg	77.0 ± 5.4	94.1 ± 11.1	0.156
Folate, μg	430.6 ± 18.1	485.8 ± 37.1	0.169
Ca^++^, mg	727.2 ± 25.3	779.2 ± 52.0	0.355
Mg^++^, mg	223.1 ± 6.3	265.5 ± 12.9	0.003
Zn^++^, mg	8.3 ± 0.3	9.5 ± 0.5	0.037
Cu^++^, mg	0.89 ± 0.02	1.02 ± 0.05	0.020
Mn^++^, mg	3.1 ± 0.1	3.8 ± 0.3	0.014
Phosphorous, mg	961.3 ± 21.0	1083.9 ± 43.2	0.009
Choline, mg	267.4 ± 8.4	295.0 ± 17.3	0.141

Data are covariate-adjusted mean ± SE. Covariates included age group, sex, BMI, total number of medical conditions, and energy intake. *p*-values for mean differences in the individual macro- and micronutrients between fatigability categories after adjusting for covariates. RAE: retinol activity equivalents; AT: alpha-tocopherol equivalents.

## Data Availability

The data presented in this study are available on request from the corresponding author.

## Supplementary Materials

The following are available online at https://www.mdpi.com/article/10.3390/geriatrics6020041/s1, Table S1: Demographic and Health-Related Characteristics for the 1201 Oldest-Old Participants of the Geisinger Rural Aging Study who Passed the Initial Screening and were Eligible for Consent Stratified by those who Completed the Study vs. Those Who Did Not Complete the Study. Table S2: Unadjusted Macro- and Micronutrient Intake Data in a Subset of 122 Oldest-Old Participants from the Geisinger Rural Aging Study According to Perceived Physical Fatigability Status: Pittsburgh Fatigability Scale (PFS).
