# Supplementary material for: The Association between Poor Diet Quality, Physical Fatigability and Physical Function in the Oldest-Old from the Geisinger Rural Aging Study
Source: Geriatrics (Basel). 2021 Apr 15;6(2):41. doi: 10.3390/geriatrics6020041 (PMC8167721; doi:10.3390/geriatrics6020041)
Supplement: Supplementary file 1 [file geriatrics-06-00041-s001.zip › geriatrics-1141159-supplementary.pdf]

Supplemental Table 1. Demographic and Health-Related Characteristics for the 1201 Oldest-Old Participants of the Geisinger Rural Aging Study who Passed the Initial Screening and were Eligible for Consent Stratified by those who Completed the Study vs. Those Who Did Not Complete the Study.

Characteristics	Potentially Eligible Participants	Eligible But Did Not Complete Study	Completed Study Participants	p-value
N	1201	1079	122	
Age				
-80-84 years, %	29.3	27.9	41.8	0.0057
-85-89 years, %	49.8	50.7	41.8	
-90+ years, %	21.0	21.5	16.4	
Female, %	57.0	58.4	54.1	0.363
History of diabetes, %	29.6	30.0	25.4	0.289
History of coronary artery disease, %	40.9	40.5	44.3	0.423
History of hypertension, %	89.2	89.7	84.4	0.075
History of obstructive sleep apnea, %	10.9	10.5	14.8	0.151
History of depression, %	7.7	8.2	3.3	0.070
History of osteoarthritis, %	58.5	59.1	53.3	0.214
History of liver disease, %	3.6	3.9	0.8	0.117
Body Mass Index (BMI), kg/m^2*	27.4 (5.0)	27.3 (5.0)	28.5 (4.9)	0.012

* Mean (SD)

Supplemental Table 2. Unadjusted Macro- and Micronutrient Intake Data in a Subset of 122 Oldest-Old Participants from the Geisinger Rural Aging Study According to Perceived Physical Fatigability Status: Pittsburgh Fatigability Scale (PFS).

Characteristics	Physical Fatigability Status		
	More, ≥15	Less, <15	p-value
N	98	24	-
Energy Intake, kcals	1452 ± 388	1580 ± 501	0.250
Fat, g	54.8 ± 19.7	58.0 ± 20.9	0.497
Carbohydrates, g	189 ± 55	205 ± 70	0.287
Protein, g	56 ± 16	68 ± 25	0.043
Protein, %	15.7 ± 3.1	17.3 ± 4.1	0.085
Protein, g/kg	0.77 ± 0.2	0.89 ± 0.4	0.139
Fiber, g	16.1 ± 5.0	20.4 ± 9.7	0.045
Vit A, µg RAE	656 ±285	823 ± 437	0.086
Vit D, µg	4.7 ± 3.1	5.8 ± 4.1	0.207
Vit E, mg AT	8.2 ± 5.3	9.6 ± 5.7	0.285
Vit K, µg	76 ± 49	144 ± 225	0.156
Vit B_6, mg	1.5 ± 0.5	2.0 ± 0.9	0.026
Vit C, mg	76 ± 44	97 ± 75	0.208
Folate, µg	435 ± 168	520 ± 236	0.108
Ca^{++}, mg	701 ± 288	810 ± 383	0.202
Mg^{++}, mg	218 ± 66	276 ± 111	0.021
Zn^{++}, mg	8.2 ± 2.5	9.9 ± 4.6	0.097
Cu^{++}, mg	0.9 ± 0.3	1.1 ± 0.4	0.049
Mn^{++}, mg	3.0 ± 1.2	3.8 ± 1.7	0.053
Phosphorous, mg	938 ± 286	1126 ± 410	0.043
Choline, mg	267 ± 96	308 ± 117	0.122

Data are Mean ± SD. p-values for unpaired T-Tests assuming unequal variances between fatigability categories. RAE: retinol activity equivalents; AT: alpha-tocopherol equivalents.
